# Expression and localization of TASK-1, -2 and -3 channels in MG63 human osteoblast-like cells

**DOI:** 10.3892/ol.2012.1088

**Published:** 2012-12-20

**Authors:** XIANTAO LI, XUEHAI DONG, SHOUCHAO ZHENG, JUN XIAO

**Affiliations:** 1 College of Biomedical Engineering, South-Central University for Nationalities, Wuhan 430074;; 2 Tongji Hospital, Tongji Medical College, Huazhong University of Science and Technology, Wuhan 430030, P.R. China

**Keywords:** acid-sensitive TASK channels, expression, cell proliferation, MG63 cells

## Abstract

It is well known that a number of ion channels are involved in the proliferation, migration and invasion of tumor cells. TASK channels, an acid-sensitive subgroup of the two-pore-domain K^+^ channel (K2P) family, are expressed in numerous types of tissue and exhibit various physiological functions depending on the cell type. In the present study, we employed RT-PCR and western blot analysis to determine the expression of TASK-1, -2 and -3 at the mRNA and protein levels in MG63 human osteoblast-like cells. Immunofluorescence with specific antibodies against the TASK channels revealed the localization patterns at the plasma membrane and the juxtanuclear compartment. The induced fluctuations in the extracellular pH from 7.4 to 6.9 and to 6.4 significantly reduced the proliferation rate of MG63 cells by 44.3 and 90.1%, respectively. These data revealed the expression of TASK-1, -2 and -3, and the correlation between TASK channels and cell proliferation in MG63 cells, suggesting that these channels may be involved in the tumorigenesis of osteosarcoma.

## Introduction

Osteosarcoma is the most frequent pediatric and adult primary bone tumor, and the majority of such tumors are malignant and tend to develop pulmonary metastases (1). Although there have been extensive efforts to explore its pathogenesis, the exact underlying mechanism of this disease remains unclear. Previous studies have suggested there are a wide range of genetic and molecular alterations in osteosarcoma, including the activation of oncogenes, the inactivation of tumor suppressor genes and the deregulation of signaling pathways (2,3). It has been demonstrated that a number of ion channels are involved in the proliferation, migration and invasion of cancer cells (4). Cancer cells are, on average, more depolarized than healthy cells of the same histological origin (5,6). In general, K^+^ channels are a critical determinant of cell membrane potential and numerous data have confirmed the presence of K^+^ channels and related currents in tumor cells (4,7). Thus, K^+^ channels may contribute to tumorigenesis by regulating the membrane potential and subsequently triggering cell proliferation.

It has been demonstrated that there are several K^+^ channels in osteoblasts, including voltage-gated K^+^ (Kv) channels (8–10), inwardly rectifying K^+^ (Kir) channels (10), ATP-sensitive K^+^ channels (11), calcium-activated K^+^ channels (12–14) and two-pore-domain K^+^ (K2P) channels (15). K2P channels are a novel family of mammalian K^+^ channels identified in the mid-to-late 1990s, which contain four trans-membrane segments and two pore domains (16–17). These channels are open at the resting membrane potential and are considered to be leak or background K^+^ channels. Their electrophysiological activity has indicated that K2P channels are able to modulate both the resting membrane potential and the action potential in different tissues, including cardiomyocytes and neurons (18–20). TREK-1, a member of the K2P channel family, contributes to the resting membrane potential of human osteoblast cells and has been observed to be correlated with the proliferation of MG63 cells (14). TASK channels, the other important subgroup of the K2P channel family have been demonstrated to be ubiquitously expressed in a number of types of tissue (21) and to also participate in the development of certain types of cancer, such as breast and lung cancer (22,23). However, little is presently known regarding the expression and possible function of TASK channels in osteoblast cells. In the present study, we set out to detect the expression of TASK channels using reverse transcription (RT)-PCR and western blot analysis in MG63 human osteoblast-like cells. Immunofluorescence was employed to reveal the localization patterns of the channels. The possibility of a role of TASK channels in the proliferation of MG63 cells was also explored. Our results present a possible alternative mechanism for the tumorigenesis of osteosarcoma.

## Materials and methods

### Cell culture

MG63 human osteoblast-like cells were cultured in RPMI-1640 medium, supplemented with 10% fetal calf serum, 100 U/ml penicillin and 100 *μ*g/ml streptomycin at 37°C in 95% humidified air with 5% CO_2_. The medium was replaced every 2–3 days, and confluent cells were digested with 0.25% trypsin and split at a rate of 1:2–1:3 every 4 days.

### Cell proliferation assay

Cell proliferation was assessed using a 2-(2-methoxy-4-nitrophenyl)-3-(4-nitrophenyl)-5-(2,4-disulfophenyl)-2H-tetrazolium (WST-8) assay (Beyotime Institute of Biotechnology, Jiangsu, China) according to the manufacturer’s instructions. Following suspension of MG63 cells in RPMI-1640 medium without phenol red, cells were transferred into 96-well plates at a density of 2×10^3^/well and incubated for 24 h. Subsequently, the medium in each well was replaced by media with different pH values (6.4, 6.9, 7.4 and 7.9) and cells were grown for 12, 24 and 48 h prior to the addition of 10 *μ*l WST-8 assay reagents. MG63 cells were subsequently incubated at 37°C for 4 h and the absorbance at 450 nm was measured using a microplate reader. All assays were performed in quintuplicate.

### PCR analysis

Total RNA was prepared from MG63 cells using RNA-Solv^®^ Reagent (Omega Bio-Tek, Inc., Norcross, GA, USA) and DNaseI (Invitrogen Life Technologies, Carlsbad, CA, USA). Total MG63 RNA (1 *μ*g) was reverse transcribed (RT) with oligo(dT) and M-MLV reverse transcriptase (Invitrogen Life Technologies).

The sense and antisense PCR oligonucleotide primers selected to amplify the cDNA are presented in Table I. The first-strand cDNA reaction mixture (1 *μ*l) was added to a 25-*μ*l PCR mixture consisting of 0.4 nM of each primer, 10 mM Tris-HCl, 50 mM KCl, 1.5 mM MgCl_2_, 200 *μ*M of each dNTP and 0.625 units of *Taq* DNA polymerase. The PCR conditions were 95°C for 5 min, followed by 35 cycles at 95°C for 30 sec, 60°C for 30 sec, 72°C for 30 sec and 72°C for 10 min. PCR products were visualized on 1.5% agarose gels stained with 0.05 *μ*g/ml GoldView (Geneshun Biotech Ltd., Guangzhou, China) and scanned using a MultiImager (Bio-Rad, Philadelphia, PA, USA).

### Immunofluorescence

For the immunofluorescence, MG63 cells were resuspended and plated onto coverslips in 22-mm Petri dishes. At 60–80% confluence, cells were fixed in 4% paraformaldehyde in phosphate-buffered saline (PBS) solution for 15 min at 4°C. Following washing three times with PBS, cells were permeated with 0.1% Triton X-100 in PBS for 10 min and then incubated with 5% BSA for 1 h at room temperature to block non-specific antibody binding. Subsequently, cells were incubated with primary antibodies, including TASK-1, -2 and -3 (1:200 dilutions; Santa Cruz Biotechnology, Inc., Santa Cruz, CA, USA), overnight at 4°C. Immunoreactivity was visualized using a donkey anti-goat IgG secondary antibody conjugated to Cy-3 (1:1,000 dilution; Jackson ImmunoResearch Laboratories, Inc., West Grove, PA, USA). Primary and secondary antibodies were diluted in 1% BSA. Coverslips were mounted using Vectashield mounting medium. Negative control experiments were performed as described previously, with the elimination of primary antibodies. Staining with 4′,6-diamidino-2-phenylindole (DAPI) was performed to highlight the cell nuclei. Images were captured using a charge-coupled device (CCD) camera connected to an inverted microscope (Olympus IX70).

### Western blot analysis

Cultured MG63 cells were rinsed three times with PBS and then lysed in RIPA buffer (50 mM Tris, pH 7.4; 1% Triton X-100; 1% sodium deoxycholate; 150 mM NaCl; 0.1% SDS and 1 mM PMSF; Beyotime Institute of Biotechnology) for 30 min on ice. Nuclei and cell debris were pelleted by low-speed centrifugation at 1,000 × g for 3–5 min at 4°C. The resulting supernatant was used as the membrane fraction. Likewise, rat cardiac muscular tissues were extracted for their proteins as positive controls by the method described previously. Proteins were then separated by 10% SDS-PAGE and transferred to a polyvinylidenefluo-ride (PVDF) membrane. Membranes were immunoblotted with antibodies against TASK-1, -2 and -3 (1:100 dilutions, Santa Cruz Biotchnology, Inc.). Horseradish peroxidaseconjugated donkey anti-goat IgG (1:3,000 dilutions; Santa Cruz Biotechnology, Inc.) was added for 1 h at room temperature for visualization.

### Statistical analysis

Results are expressed as mean ± standard error. Statistical significance was calculated using a one-way analysis of variance (ANOVA) and P<0.05 was considered to indicate a statistically significant difference.

## Results

### mRNA expression of TASK channels in MG63 cells

**Figure 1:**
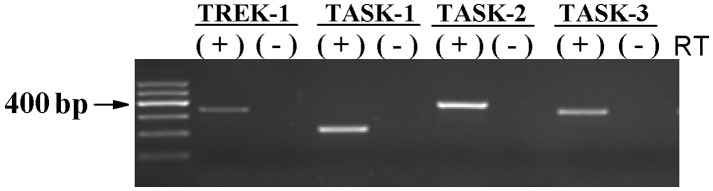
mRNA expression of TASK channels. The PCR product of TREK-1 (340 bp) in MG63 cells was used as a positive control. MG63 cells displayed 221-, 388- and 337-bp bands for TASK-1, -2 and -3, respectively, corresponding to the expected lengths. RT (+) indicates a reaction with reverse trans criptase and RT (−) indicates the omission of reverse trans criptase from the reaction. The first lane shows the DNA maker.

Among the subfamily of acid-sensitive TASK channels, TASK-1, -2 and -3 are functionally expressed in various types of tissue and thus these channels have been intensely investigated (24). Therefore, we only designed primers and performed RT-PCR to investigate the mRNA transcripts of TASK-1, -2 and -3 in MG63 human osteoblast-like cells in the present experiments (Table I). The PCR products of the predicted size for TASK-1 (221 bp), TASK-2 (388 bp) and TASK-3 (337 bp) were amplified from total RNA templates of MG63 cells (Fig. 1; n=4). Hughes *et al* previously observed the expression of TREK-1, the other member of the K2P family, in human MG63 cells (15). Therefore, the PCR product of TREK-1 was also detected at the expected size (340 bp) and used to validate RT (Fig. 1, left). No signals were present in the RT (−) which omitted reverse transcriptase from the reaction in repeated experiments, suggesting that this is not a contaminant of genomic DNA.

### Detection of TASK channel protein in MG63 cells

**Figure 2:**
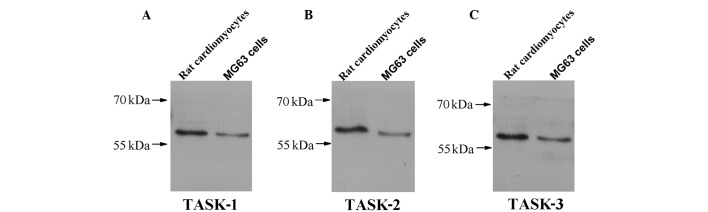
Western blot analysis for TASK channels in MG63 cells. The molecular weight is indicated on the left side of the gel. Rat cardiomyocytes were used as positive control samples for TREK-1. The expected size bands were detected for (A) TASK-1, (B) TASK-2 and (C) TASK-3.

Although RT-PCR confirmed that TASK-1, -2 and -3 mRNA was present in MG63 cells, it did not necessarily indicate that the mRNA was translated into protein. The expression of TASK channels at the protein level was further explored by western blot analysis. Western blot analysis of whole cell protein extracts revealed the presence of one band at 60 kDa for TASK-1, 58 kDa for TASK-2 and 57 kDa for TASK-3 (Fig. 2; n=3). A previous study demonstrated that all three TASK channels were expressed in cardiomyocytes (19). Thus, the protein signals of these channels in rat cardiac tissues were used as positive controls (Fig. 2).

### Immunofluorescence of TASK-1, -2 and -3 in MG63 cells

**Figure 3:**
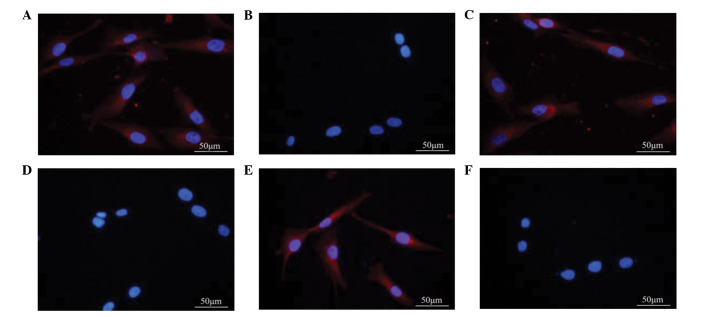
Immunofluorescence of TASK channels in MG63 cells. (A, C and E) Immunostaining images for TASK-1, -2 and -3 proteins (red), respectively. (B, D and F) Staining was absent when anti-TASK-1, -2 and -3 antibodies were omitted. Staining with 4′,6-diamidino-2-phenylindole (DAPI) highlighted the cell nuclei (blue). The scale bar in all panels is 50 *μ*m.

Immunocytochemical staining with specific antibodies indicated that TASK-1, -2 and -3 exhibited the localization patterns of the plasma membrane and the juxtanuclear compartment in MG63 cells (Fig. 3A, C and E). Staining of the TASK channels was strongest around the nuclei and relatively weak along cell processes. Hughes *et al* demonstrated that TREK-1 displayed similar localization patterns comapred with the present study in MG63 cells (14). When the primary antibodies were omitted from the experiments, staining of the TASK channels was not observed in the images (Fig. 3B, D and F). The cell nuclei are shown with DAPI staining (blue).

### Extracellular acidosis affected cell proliferation

**Figure 4:**
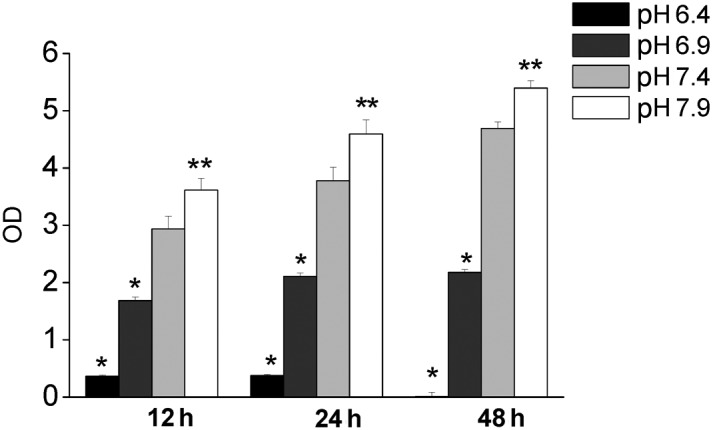
Effects of extracellular acidification on cell proliferation. The graph shows that the different pH values altered the proliferation rate of MG63 cells (n=5). ^*^P<0.05 and ^**^P>0.05 compared with pH 7.40.

TASK channels have also been demonstrated to participate in the development of certain types of cancer, including breast and lung cancer (23,24). In the present study, we identified expression of TASK-1, -2 and -3 at the mRNA and protein levels in MG63 cells. To explore the involvement of TASK channels in the tumorigenesis of osteosarcoma, further experiments were designed to investigate the correlation between cell proliferation and TASK channels in MG63 cells. It is well known that TASK-1, -2 and -3 are acid-sensitive channels and are inhibited by extracellular acidosis. Thus, MG63 cells were cultured in a medium with different pH values (6.4, 6.9, 7.4 and 7.9) for 12, 24 and 48 h, and cell proliferation was subsequently measured by a WST-8 as described in Materials and methods. In contrast to pH 7.4, extracellular acidosis at pH 6.9 and 6.4 significantly reduced the proliferation of MG63 cells, without time dependence, by 44.3 and 90.1%, respectively (Fig. 4; n=5, P<0.05). However, no effects were observed in the medium with a pH value of 7.9 (Fig. 4; n=5, P>0.05). Therefore, extra-cellular acidosis may regulate cell proliferation by inhibiting the TASK channels.

## Discussion

The K2P channel is a background or leak channel, which is widely distributed in both excited and unexcited cells, where it has important physiological roles (24). In the present study, to the best of our knowledge, we identified for the first time the presence of acid-sensitive TASK-1, -2 and -3 channels in MG63 human osteoblast-like cells. The distinct mRNA and protein expression levels of these channels were detected by RT-PCR and western blot analysis, respectively. In addition, immunofluorescence with specific antibodies against the three TASK channels revealed localization at the plasma membrane and the juxtanuclear compartment in MG63 cells.

The TREK-1 channel, a mechanosensitive member of the K2P channel family, has been observed to be functionally expressed in MG63 cells and to participate in setting the resting membrane potential of these cells (15). Moreover, blockages of TREK-1 by bupivacaine have been demonstrated to upregulate bone cell proliferation (15). Previous studies have suggested that TASK channels, an acid-sensitive subgroup of the K2P channel family, are involved in the development of certain types of cancer, including breast and lung cancer (22,23). Thus, it is reasonable to speculate that TASK channels may have possible roles in the pathogenesis of tumors. Our related experiments have demonstrated that cell proliferation is regulated by extracellular acidosis and is correlated with TASK channels.

Various causes, such as renal or lung disease, are capable of leading to tissue acidosis. The negative impact of acidosis on the skeleton has been known to result from physicochemical dissolution of bone mineral. For rapidly growing tumors, the shortage of the vascular supply has been demonstrated to reduce perfusion and resulted in ischemia and hypoxia (25); in turn, local acidification occurred and may inhibit acid-sensitive TASK channels. Brandao-Burch *et al* reported that the proliferation of rat primary osteoblast was unaffected by pH in the range 7.4-6.9 (26); however, we found that pH 6.9 significantly reduced the proliferation of MG63 cells. The exact reason for the difference is unclear and the diversity of the species may be one possible cause. Notably, cell proliferation was downregulated, but not upregulated, by the inhibition of TASK channels compared with the blockage of TREK-1 in MG63 cells. The two subfamilies of K2P channels, TREK and TASK, may therefore exert opposite effects on the proliferation of MG63 cells.

Lauritzen *et al* demonstrated that extracellular acidosis prevented the neuronal death of cultured rat granule neurons by reducing K^+^ efflux through the TASK-3 channels (27). In the present study, the death of MG63 human osteoblast-like cells induced by extracellular acidosis was at least partially due to the inhibition of TASK channels. Thus, TASK channels exert either protective or deteriorative effects depending on the cell type. It is well known that tumor cells are more depolarized than healthy cells, and that membrane depolarization improves the proliferation rate of tumor cells (5,6). Nevertheless, our data suggested that the inhibition of TASK channels by extracellular acidosis induced depolarization and subsequently prevented cell proliferation. The apparent contradiction implies that our understanding of the correlation between ion channels and tumorigenesis is poor.

A limitation of the present study is that extracellular acidification may not only act on TASK channels, but also on other types of ion channels, including VR1, ASIC and NMDA, which so far have not been identified in MG63 cells. Thus, we are not able to completely exclude the possible effects of extra-cellular acidosis on other acid-sensitive channels. Moreover, although the apoptosis rate of cultured rat primary osteoblasts was barely affected by pH in the range 7.4-6.9 (26), further experiments ought to be performed to clarify the effects of acidosis on the apoptosis of MG63 cells.

In summary, the data from the RT-PCR and western blot analysis demonstrated for the first time that TASK-1, -2 and -3 were expressed in MG63 cells, while immunofluorescence revealed the localization patterns of these channels. Extracellular acidosis exerted an inhibitory effect on the proliferation rate of MG63 cells, indicating that acid-sensitive TASK channels are correlated with cell proliferation.

## Figures and Tables

**Table I:** Oligonucleotide sequences of primers used for reverse transcription-polymerase chain reaction (RT-PCR).

Gene	GI	Forward primer	Reverse primer	Length (bp)
TREK-1	5712620	GTGGAATGTTAGTCAGACCAAG	TCTGAACTCTCCCACCTCTTC	340
TASK-1	197245365	GTGCTCATCGGCTTCTTCTC	GTGAGGCCCGTAAGGATGTA	221
TASK-2	88999598	TGGACAAGATCCTAGAGGTGGT	TCAGTCACCATGAATACGAAGG	388
TASK-3	16445406	GACCATGAACAGTGAGGATGAG	GTGTAACCCAGGAGAGATGGAG	337

TREK-1, a lipid-sensitive member of the K2P channel family; TASK, acid-sensitive members of the K2P channel family; GI, GenInfo Identifier sequence ID provided by the National Center for Biotechnology Information.
